# Provider‐to‐provider telemedicine for sepsis is used less frequently in communities with high social vulnerability

**DOI:** 10.1111/jrh.12861

**Published:** 2024-06-26

**Authors:** Kevin J. Tu, J. Priyanka Vakkalanka, Uche E. Okoro, Karisa K. Harland, Cole Wymore, Brian M. Fuller, Kalyn Campbell, Morgan B. Swanson, Edith A. Parker, Luke J. Mack, Amanda Bell, Katie DeJong, Brett Faine, Anne Zepeski, Keith Mueller, Elizabeth Chrischilles, Christopher R. Carpenter, Michael P. Jones, Marcia M. Ward, Nicholas M. Mohr

**Affiliations:** ^1^ Department of Cell Biology and Molecular Genetics University of Maryland College Park Maryland USA; ^2^ University of Maryland School of Medicine Baltimore Maryland USA; ^3^ Department of Emergency Medicine University of Iowa Carver College of Medicine Iowa City Iowa USA; ^4^ Cancer Research UK Cambridge Institute University of Cambridge Cambridge UK; ^5^ Department of Epidemiology University of Iowa College of Public Health Iowa City Iowa USA; ^6^ University of Iowa Carver College of Medicine Iowa City Iowa USA; ^7^ Division of Critical Care Department of Anesthesiology Washington University School of Medicine St. Louis Missouri USA; ^8^ Department of Emergency Medicine Washington University School of Medicine St. Louis Missouri USA; ^9^ Department of Surgery Hennepin County Medical Center Minneapolis Minnesota USA; ^10^ Department of Community & Behavioral Health University of Iowa College of Public Health Iowa City Iowa USA; ^11^ Avel eCARE Sioux Falls South Dakota USA; ^12^ Department of Family Medicine University of South Dakota School of Medicine Sioux Falls South Dakota USA; ^13^ Department of Pharmacy Practice & Science College of Pharmacy University of Iowa Iowa City Iowa USA; ^14^ Department of Pharmaceutical Care University of Iowa Hospitals & Clinics Iowa City Iowa USA; ^15^ Department of Health Management and Policy University of Iowa Hospitals & Clinics Iowa City Iowa USA; ^16^ Department of Health Management and Policy University of Iowa College of Public Health Iowa City Iowa USA; ^17^ Department of Emergency Medicine Mayo Clinic Rochester Minnesota USA; ^18^ Department of Biostatistics University of Iowa College of Public Health Iowa City Iowa USA; ^19^ Division of Critical Care Department of Anesthesia University of Iowa Carver College of Medicine Iowa City Iowa USA

**Keywords:** critical care, emergency medicine, health inequities, sepsis, social vulnerability, telemedicine

## Abstract

**Purpose:**

Sepsis disproportionately affects patients in rural and socially vulnerable communities. A promising strategy to address this disparity is provider‐to‐provider emergency department (ED)‐based telehealth consultation (tele‐ED). The objective of this study was to determine if county‐level social vulnerability index (SVI) was associated with tele‐ED use for sepsis and, if so, which SVI elements were most strongly associated.

**Methods:**

We used data from the TELEmedicine as a Virtual Intervention for Sepsis in Rural Emergency Department study. The primary exposures were SVI aggregate and component scores. We used multivariable generalized estimating equations to model the association between SVI and tele‐ED use.

**Findings:**

Our study cohort included 1191 patients treated in 23 Midwestern rural EDs between August 2016 and June 2019, of whom 326 (27.4%) were treated with tele‐ED. Providers in counties with a high SVI were less likely to use tele‐ED (adjusted odds ratio [aOR] = 0.51, 95% confidence interval [CI] 0.31‒0.87), an effect principally attributable to the housing type and transportation component of SVI (aOR = 0.44, 95% CI 0.22–0.89). Providers who treated fewer sepsis patients (1‒10 vs. 31+ over study period) and therefore may have been less experienced in sepsis care, were more likely to activate tele‐ED (aOR = 3.91, 95% CI 2.08‒7.38).

**Conclusions:**

Tele‐ED use for sepsis was lower in socially vulnerable counties and higher among providers who treated fewer sepsis patients. These findings suggest that while tele‐ED increases access to specialized care, it may not completely ameliorate sepsis disparities due to its less frequent use in socially vulnerable communities.

## INTRODUCTION

Sepsis is a systemic inflammatory response to infection that causes organ injury and mortality in 15%‒25% of cases.^1^ Sepsis affects over 1.7 million Americans annually, contributing to over $41 billion in hospital expenses.^2^, ^3^ Sepsis mortality disproportionately affects rural patients, as sepsis mortality decreases up to 36% as the emergency department (ED) case volume increases.^4^, ^5^ This trend is likely due to less variable sepsis guideline adherence, fewer staffing challenges, shorter distances to receive care, and more streamlined processes of care in higher volume EDs.^5^, ^6^, ^7^, ^8^

Social vulnerability refers to the susceptibility of populations to adverse health outcomes due to underlying socioeconomic, demographic, and environmental factors. The social vulnerability index (SVI) is calculated using 16 factors, which are grouped into four dimensions: (1) socioeconomic status, (2) household composition and disability, (3) minority status and language, and (4) housing type and transportation.^9^ Social disadvantage, and by extension vulnerability, has been associated with increased health disparities for many populations, including within sepsis.^10^, ^11^ Rurality is related to social vulnerability, and addressing sepsis outcome disparities may reduce the overall burden of sepsis‐related mortality and economic costs.^12^

Methods to improve rural sepsis outcomes, which promote early diagnosis and guideline adherence, may provide opportunities for reducing sepsis disparities.^13^ One promising strategy is the implementation of provider‐to‐provider ED‐based telehealth consultation (tele‐ED). Rural EDs have less specialized care than urban EDs; tele‐ED helps overcome this by connecting rural providers with board‐certified emergency physicians to promote guidance for early sepsis care and to arrange interhospital transfer.^7^, ^14^, ^15^ However, the benefit and use of tele‐ED is highly context dependent.^13^ For example, tele‐ED was associated with lower sepsis mortality when activated early by the rural provider, used by rural advanced practice providers (APPs), or employed in hospitals with higher baseline sepsis mortality.^13^, ^16^ Tele‐ED improved adherence to sepsis bundle care, a set of guidelines designed to improve sepsis outcomes, in rural community EDs, but this finding has not been universal.^16^, ^17^, ^18^

The objective of this study was to determine if county‐level social vulnerability was associated with increased tele‐ED use for sepsis and, if so, which elements of social vulnerability were most strongly associated. Our findings will help determine to what extent tele‐ED can be used to narrow disparities in sepsis care and how it can be more effectively harnessed to reduce outcome disparities.

## METHODS

### Study design and patient inclusion

We used data from TELEmedicine as a Virtual Intervention for Sepsis in Emergency Departments (TELEVISED, ClinicalTrials.gov NCT04441944), a retrospective cohort study that measured the association between tele‐ED use in sepsis care and outcomes in a mature, rural tele‐ED network.^16^, ^19^ Patient selection is described in the original TELEVISED studies.^16^, ^19^ Briefly, TELEVISED involved adult (≥18 years of age) sepsis patients from 23 rural acute hospitals in the upper Midwest of the United States between August 1, 2016 and June 30, 2019. We defined rural as a rural‒urban continuum code (RUCC) index >3 and 99.6% of patients were treated in a community with an RUCC index >7. One hospital in our cohort was from a county with an RUCC index 3. However, we made an exception to this hospital as it was a designated critical access hospital. Sepsis was defined as meeting all of the following criteria: (1) inpatient discharge diagnosis of sepsis based on International Classification of Diseases, 10th edition, Clinical Modification codes, (2) infection diagnosed in the ED, (3) sequential organ‐failure assessment score of at least 2, and (4) meeting at least two systemic inflammatory response syndrome criteria.^16^, ^19^ The tele‐ED intervention included standardized nurse‐directed sepsis screening and tele‐ED consultation used at the discretion of the local treating clinician. Tele‐ED care, available 24 hours a day, involved a high‐definition video on‐demand connection with a tele‐ED hub located in Sioux Falls, SD, where a board‐certified emergency physician and experienced ED nurse provided consultation with local staff. The study was approved by the local institutional review boards of participating centers and is reported to be consistent with the Template for Intervention Description and Replication checklist for Population Health and Policy and the Strengthening the Reporting of Observational Studies in Epidemiology statements.^20^, ^21^

### Patient, provider, hospital, and community variables

We captured social vulnerability of each county (based on the hospital location) using the Centers for Disease Control and Prevention's nationally normalized social vulnerability index (SVI) from 2016 to 2018.^9^ A higher SVI represents greater social vulnerability relative to other communities in the country. Patient demographic factors and hospital‐level characteristics were abstracted from electronic medical records.^16^, ^19^ Provider‐level factors included the number of sepsis patients (1‒10, 11‒31, 31+) treated by a given provider during the study period and provider type (APP alone, physician alone, or an APP with on‐site physician). APPs were defined as healthcare professionals who have the authority to diagnose, treat, and manage patient care but who are not physicians. Low‐volume EDs were defined as having less than 7500 patients annually. We linked community‐level variables with the hospital city using data from the 2016 and 2018 American Community Survey (ACS) 5‐year estimates,^22^ 2013 RUCC, and 2016 and 2018 medically underserved area dataset (MUA).^23^ Community‐level factors included city population (ACS), median county age (ACS), percent nonwhite population (ACS), percent below poverty level (ACS), median household income (ACS), RUCC, and MUA status. Variables collected from 2016 and 2018 were matched to patients treated in 2016‒2017 and 2018‒2019, respectively.

### Outcomes

The outcome in this study was tele‐ED use, defined as using video tele‐ED during the ED visit for a patient with sepsis. During consultations, the tele‐ED hub also used computerized real‐time decision‐support software to guide recommendations and facilitate documentation for Surviving Sepsis Campaign guideline‐adherent sepsis patient care. Sepsis cases identified at the local hospital with a matching visit in the tele‐ED hub call log were classified as tele‐ED exposed.

### Statistical analysis

We tabulated patient, provider, hospital, and community measures, stratified by tele‐ED utilization. Continuous county‐level variables (city population, median age, percent nonwhite, percent below poverty level, median household income, and SVI scores) were categorized as above/below the median values of the counties for the sample hospitals in the dataset. Since there were multiple levels of nesting (patients, providers, EDs, and county), we initially developed generalized linear mixed models to account for the nested levels. However, as we evaluated the distribution of units within each level, we discovered that many providers saw only one patient and several counties had only one hospital, so that multi‐level (four levels) modeling with level‐specific covariates was not practical. As such a single level of clustering was sufficient and the most relevant clustering was patient within hospital ED. We then evaluated the unadjusted odds ratio (uOR) and 95% confidence intervals (CIs) between each covariate and tele‐ED utilization through generalized estimating equations with a logit link and binomial distribution. We developed multivariable models predicting tele‐ED use with covariates at each stratum (i.e., patient, provider, ED/hospital, and county) and identified the most parsimonious models using a backward elimination procedure guided by the quasi‐likelihood under the Independence Model Criterion (QIC).^20^, ^21^

We screened for collinearity and significance of interaction terms. SVI was the county‐level primary exposure of interest. We developed two models, one for the overall SVI score and one for the SVI component scores only. All analyses were completed using SAS v9.4 (SAS Institute), and statistical significance was defined as *p* < 0.05 using two‐tailed tests.

## RESULTS

### Patient, provider, hospital, and community‐level characteristics

**FIGURE 1:**
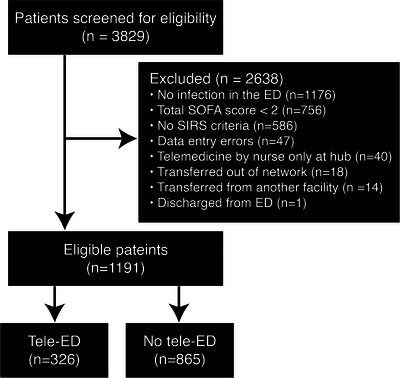
Flowchart of patient inclusion. ED, emergency department; SIRS, systemic inflammatory response syndrome; SOFA, sequential organ failure assessment.

Our study cohort included 1191 patients, of whom 326 (27.4%) were treated with tele‐ED (Figure 1).^16^ The median age was 72 years (interquartile range [IQR] 62‒82 years), 535 (44.9%) were women, and 1059 (88.9%) patients were white (Table 1). Tele‐ED was more likely to be used for patients who were male (uOR = 1.30, 95% CI 1.09–1.56) and between the ages of 45 and 64 years (uOR = 1.91, 95% CI 1.41–2.58). At the provider level, the study included 193 providers across 23 hospitals in Iowa, Nebraska, South Dakota, and Minnesota. APPs (uOR = 5.14, 95% CI 2.45–10.79) and those who saw fewer sepsis patients (1–10 vs. 31+, uOR = 4.01, 95% CI 2.46–6.52) were more likely to use tele‐ED. Low‐volume EDs (<7500 patients annually) comprised 19 (83%) of the hospitals and were associated with higher tele‐ED use (uOR = 2.80, 95% CI 1.09–7.19). Tele‐ED was more likely to be used in medically underserved counties (uOR = 1.88, 95% CI 0.84–4.20).

**TABLE 1:** Characteristics grouped by telemedicine use.

			Tele‐ED (*n* = 326)		No tele‐ED (*n* = 865)		Unadjusted OR^a^		
Characteristics	All (*n* = 1191)						uOR	L 95% CI	U 95% CI
**Patient characteristics**									
Sex, *n* (%)									
Male	656	55.1%	195	59.8%	461	53.3%	1.30	1.09	1.56
Female	535	44.9%	131	40.2%	404	46.7%	Ref		
Age (years), *n* (%)									
18‒44	72	6.1%	25	7.7%	47	5.4%	1.74	0.94	3.23
45‒64	288	24.2%	106	32.5%	182	21.0%	1.91	1.41	2.58
65+	799	67.1%	187	57.4%	612	70.8%	Ref		
Missing	32	2.7%	8	2.5%	24	2.8%	1.09	0.51	2.31
Race, *n* (%)									
White	1,059	88.9%	286	87.7%	773	89.4%	Ref		
African American	8	0.7%	5	1.5%	3	0.4%	4.50	2.08	9.77
American Indian	93	7.8%	27	8.3%	66	7.6%	1.11	0.40	3.07
Asian	3	0.3%	2	0.6%	1	0.1%	5.42	3.35	8.72
Missing/unknown	28	2.4%	6	1.8%	22	2.5%	0.74	0.28	1.97
Clinical characteristics									
Triage systolic pressure (mmHg), mean (SD)	130.3	29.3	126.1	29.7	131.9	29.0	0.99	0.98	0.99
SOFA score, mean (SD)	4.0	1.9	4.7	2.4	3.7	1.7	1.06	1.04	1.08
Infection source: respiratory/pneumonia, *n* (%)	636	53.4%	134	41.1%	502	58.0%	0.50	0.30	0.85
BMI category, *n* (%)									
<18.5	119	10.0%	33	10.1%	86	9.9%	0.87	0.62	1.23
18.5‒24.9	295	24.7%	68	20.9%	227	26.2%	0.68	0.51	0.91
25‒29.9	299	25.1%	79	24.2%	220	25.4%	0.82	0.63	1.06
≥30	478	40.1%	146	44.8%	332	38.4%	Ref		
Disposition									
Transferred, *n* (%)	359	30.1%	286	87.7%	73	8.4%	77.6	40.0	150.5
Local admit, *n* (%)	832	69.9%	40	12.3%	791	91.6%	Ref		
Comorbidities, *n* (%)									
Asthma	143	12.0%	40	12.3%	103	11.9%	1.03	0.65	1.64
Cancer	355	29.8%	88	27.0%	267	30.9%	0.83	0.64	1.06
Chronic dialysis	51	4.3%	29	8.9%	22	2.5%	3.74	2.08	6.75
COPD	378	31.7%	86	26.4%	292	33.8%	0.70	0.48	1.02
Cirrhosis	31	2.6%	13	4.0%	18	2.1%	1.95	1.16	3.31
Congestive heart failure	255	21.4%	71	21.8%	186	21.3%	1.03	0.83	1.28
Diabetes	443	37.2%	126	38.7%	317	36.6%	1.09	0.88	1.34
Hypertension	856	71.9	225	69.0%	631	73.0%	0.83	0.67	1.02
Solid organ transplant	12	1.0%	4	1.2%	8	0.9%	1.33	0.44	4.01
**Provider level**									
Provider type, *n* (%)									
APP alone	63	5.3%	38	11.7%	25	2.9%	5.14	2.45	10.79
APP with provider	116	9.7%	57	17.5%	59	6.8%	3.27	1.49	7.14
Physician	1,015	85.0%	231	70.9%	781	90.3%	Ref		
Sepsis patients seen by provider, *n* (%)									
1‒10	451	37.9%	194	59.5%	257	29.7%	4.01	2.46	6.52
11‒31	311	26.1%	64	19.6%	247	28.6%	1.38	0.54	3.51
31+	429	36.0%	68	20.9%	361	41.7%	Ref		
**Hospital level**									
ICU, *n* (%)									
No	546	45.8%	235	72.1%	311	36.0%	4.60	2.63	8.05
Yes	645	54.2%	91	27.9%	554	64.1%	Ref		
Annual ED volume, *n* (%)									
<7500	659	55.3%	237	72.7%	422	48.8%	2.80	1.09	7.19
7500‒14,999	532	44.7%	89	27.3%	443	51.2%	Ref		
Total inpatient beds, *n* (%)									
<50	563	47.3%	240	73.6%	323	37.3%	4.68	2.71	8.09
50‒75	628	52.7%	86	26.4%	542	62.7%	Ref		
Distance to transfer receiving hospital in miles, *n* (%)									
<60	118	9.9%	55	16.9%	63	7.3%	Ref		
60‒89	390	32.8%	60	18.4%	330	38.2%	0.21	0.08	0.56
90‒119	270	22.7%	100	30.7%	170	19.7%	0.67	0.23	2.00
>120	413	34.7%	111	34.1%	302	34.9%	0.42	0.13	1.39
**County level**									
MUA, *n* (%)									
No	443	37.2%	88	27.0%	355	41.0%	Ref		
Yes	748	62.8%	238	73.0%	510	59.0%	1.88	0.84	4.20
SVI index (overall), *n* (%)									
≤0.25	555	51.7%	196	60.1%	420	48.6%	Ref		
>0.25	636	48.3%	130	39.9%	445	51.5%	0.63	0.25	1.59
SVI socioeconomic status subscore, *n* (%)									
≤0.12	584	49.0%	166	50.9%	418	48.3%	Ref		
>0.12	607	51.0%	160	49.1%	447	51.7%	0.90	0.34	2.42
SVI household characteristics subscore, *n* (%)									
≤0.30	596	50.0%	135	41.4%	461	53.3%	Ref		
>0.30	595	50.%	191	48.6%	404	46.7%	1.94	1.60	2.35
SVI racial and ethnic minority subscore, *n* (%)									
≤0.35	497	41.7%	143	43.9%	354	40.9%	Ref		
>0.35	694	58.3%	183	56.1%	511	59.1%	0.89	0.33	2.39
SVI housing type and transportation subscore, *n* (%)									
≤0.5	498	41.8%	213	65.3%	285	33.0%	Ref		
>0.5	693	58.2%	113	34.7%	580	67.1%	0.26	0.14	0.50
Local population, *n* (%)									
≤10,000	546	45.8%	235	72.1%	311	36.0%	Ref		
>10,000	645	54.2%	91	27.9%	554	64.1%	0.22	0.12	0.38
Median age (years), *n* (%)									
≤40	789	66.3%	156	66.3%	633	73.2%	Ref		
>40	402	33.8%	170	33.8%	232	26.8%	2.97	1.27	6.95
Percent of nonwhite population, *n* (%)									
≤10%	815	68.4%	228	69.9%	587	67.9%	Ref		
>10%	376	31.6%	98	30.1%	278	32.1%	0.91	0.32	2.59
Percent below poverty level, *n* (%)									
≤10%	351	29.5%	126	38.7%	225	26.0%	Ref		
>10%	840	70.5%	200	61.4%	640	74.0%	0.56	0.25	1.23
Median household income, *n* (%)									
≤$50,000	526	44.2%	136	41.7%	390	45.1%	Ref		
>$50,000	665	55.8%	190	58.3%	475	54.9%	1.15	0.42	3.11

*Note*: County‐level measures were categorized at/around the median for the hospitals in the sample.

Abbreviations: APP, advanced practice provider; BMI, body mass index; CI, confidence interval; COPD, chronic obstructive pulmonary disease; ED, emergency department; ICU, intensive care unit; MUA, medically underserved area; OR, odds ratio; Ref, reference; SD, standard deviation; SOFA, sequential organ failure assessment; SVI, social vulnerability index; tele‐ED, ED‐based telehealth consultation; uOR, unadjusted odds ratio.

^a^ Unadjusted models include hospital site as a cluster variable. uOR for SOFA score represents the impact of a one‐unit increase in score on telemedicine utilization, while uOR for systolic blood pressure (SBP) represents a 10‐unit increase in SBP on the odds of telemedicine utilization.

### Multivariable model

Our multivariable model retained the following variables: pneumonia (patient level), number of sepsis patients seen by a provider (provider level), annual ED volume (hospital level), and SVI (county level). All other variables were dropped during variable selection. Tele‐ED use was greater among providers who treated 1–10 sepsis patients during the study period compared to those who treated 31+ patients (adjusted odds ratio [aOR] = 2.86, 95% CI 1.79–4.54) and low‐volume EDs compared to high‐volume EDs (aOR = 2.77, 95% CI 1.80–4.24) (Table 2).

**TABLE 2:** Association of aggregate social vulnerability index (SVI) and component SVI scores with emergency department (ED)‐based telehealth consultation use.

Characteristics	Aggregate SVI score, *n* = 23			Component SVI scores, *n* = 23		
	aOR	L 95% CI	U 95% CI	aOR	L 95% CI	U 95% CI
Patient level						
Pneumonia	0.51	0.30	0.86	0.52	0.31	0.89
Provider level						
Sepsis patients seen						
1‒10	2.86	1.79	4.54	1.83	0.98	3.44
11‒30	1.27	0.74	2.17	1.05	0.60	1.83
31+	Ref			Ref		
Hospital level						
Annual ED volume						
<7500	2.77	1.80	4.24	2.46	1.25	4.81
7500‒15,000	Ref			Ref		
County level						
Aggregate SVI						
High (>0.25)	0.51	0.31	0.87	–	–	–
Low (≤0.25)	Ref			–		
SVI subscore—socioeconomic status						
High subscore (>0.12)	–	–	–	0.93	0.56	1.56
Low subscore (≤0.12)	–			Ref		
SVI subscore—household characteristic status						
High subscore (>0.30)	–	–	–	0.88	0.45	1.73
Low subscore (≤0.30)	–			Ref		
SVI subscore—racial and ethnic minority status						
High subscore (>0.35)	–	–	–	1.19	0.58	2.45
Low subscore (≤0.35)	–			Ref		
SVI subscore—housing type and transportation status						
High subscore (>0.50)	–	–	–	0.44	0.22	0.89
Low subscore (≤0.50)	–			Ref		

*Note*: Model 1 uses aggregate SVI score. Model 2 uses component SVI scores.

Abbreviations: aOR, adjusted odds ratio; CI, confidence interval; Ref, reference.

High SVI was associated with significantly lower tele‐ED use (aOR = 0.51, 95% CI 0.31–0.87, Table 2). Replacing SVI with its component SVI subscores revealed that the decrease in tele‐ED use was most attributable to the community‐level housing type and transportation SVI subscore (aOR = 0.44, 95% CI 0.22–0.89, Table 2). All other SVI components were not significantly associated. [Correction added on 03 July 2024, after first online publication: An extraneous incomplete sentence was deleted from the end of this paragraph.]

## DISCUSSION

Tele‐ED was less likely to be used in rural EDs within socially vulnerable counties, an effect most strongly associated with the housing and transportation dimensions of social vulnerability. We also found that providers who saw the fewest patients and providers practicing in low‐volume EDs were the most likely to use tele‐ED.

Our results are the first to evaluate tele‐ED for sepsis care through the lens of health equity.^13^ Although tele‐ED has been posited as a method to reduce disparities in sepsis care, our data suggest similar disparities in its use that may limit its disparity‐reducing effectiveness. Previous direct‐to‐consumer telemedicine studies found that telemedicine growth is lowest in socially vulnerable communities, and our ED‐based findings seem to corroborate this same pattern.^24^, ^25^, ^26^, ^27^

We speculate that housing and transportation SVI is indicative of poorer, more remote areas. A recent qualitative study suggested that other factors affect tele‐ED use, such as the standardization of sepsis care in remote areas and real‐time experiential learning by providers who use tele‐ED.^28^ It is possible that, in being in a more remote area, the hospitals needed to adopt more standardized sepsis care bundle procedures or may be more confident with the care they were providing—confidence that stemmed from prior tele‐ED use. More broadly, there is also evidence to suggest that there may also be cultural differences that limit the adoption of tele‐ED in hospitals located in counties with high social vulnerability. In a survey of academic and rural community hospital staff, providers in critical access EDs indicated that they would use tele‐ED much less frequently than predicted by providers in academic medical centers for similar applications, despite both groups having similar perceptions of telemedicine's impact on patient outcomes.^29^ Alternatively, there may also be a lower perceived need for tele‐ED in critical access hospitals as perhaps the most remote hospitals attract particularly independent providers. Regardless, understanding providers' technology adoption in socially vulnerable communities can inform health care policy and the design of telehealth interventions. Without more targeted interventions, the continued disparity in tele‐ED use may perpetuate limited access to specialized care and exacerbate existing sepsis disparities.

As expected, we also observed that providers who treated fewer sepsis patients and therefore may be less experienced in sepsis care were more likely to activate tele‐ED. This was similar to previous findings that suggest APPs used tele‐ED to seek more guidance in sepsis care, possibly reducing mortality.^16^ This suggests that tele‐ED was effective at increasing access to specialized sepsis care in rural EDs—even though aggregate use was low. Thus, interventions that boost tele‐ED use in socially vulnerable hospitals may help reduce sepsis disparities.

Our study has limitations. Our sample size included only 23 hospitals across 22 counties in a single mature tele‐ED network, which affects our ability to interpret community‐level predictors of tele‐ED use, especially in higher volume EDs. The retrospective nature of our study also may have introduced misattribution from unmeasured confounding variables. Further understanding the contexts tele‐ED is most valuable in, especially in socially vulnerable areas, may inform the development of telehealth infrastructure and training programs that maximize tele‐ED's effect on patient outcomes and disparities.

In conclusion, our findings suggest that despite potentially increasing access to specialized care, tele‐ED was less likely to be used in EDs within socially vulnerable communities. Unless tele‐ED use is increased in socially vulnerable hospitals, tele‐ED may not be as effective in narrowing disparities in sepsis care as an optimized system might be. Emphasis on continued resource support, healthcare infrastructure development, and recruitment of ED specialists will thus be critical to address sepsis disparities.

## CONFLICT OF INTEREST STATEMENT

Luke J. Mack, Amanda Bell, and Katie DeJong are employed by Avel eCARE, which provides emergency department‐based telemedicine services. None of the other authors has any competing financial interests to report.
